# Bisgma Stimulates Prostaglandin E2 Production in Macrophages via Cyclooxygenase-2, Cytosolic Phospholipase A2, and Mitogen-Activated Protein Kinases Family

**DOI:** 10.1371/journal.pone.0082942

**Published:** 2013-12-23

**Authors:** Yu-Hsiang Kuan, Fu-Mei Huang, Shiuan-Shinn Lee, Yi-Ching Li, Yu-Chao Chang

**Affiliations:** 1 Department of Pharmacology, Chung Shan Medical University, Taichung, Taiwan; 2 Department of Dentistry, Chung Shan Medical University Hospital, Taichung, Taiwan; 3 School of Public Health, Chung Shan Medical University, Taichung, Taiwan; 4 School of Dentistry, Chung Shan Medical University, Taichung, Taiwan; China Medical University, Taiwan

## Abstract

**Background:**

Bisphenol A-glycidyl-methacrylate (BisGMA) employs as a monomer in dental resins. The leakage of BisGMA from composite resins into the peripheral environment can result in inflammation *via* macrophage activation. Prostaglandin E2 (PGE2) is a key regulator of immunopathology in inflammatory reactions. Little is known about the mechanisms of BisGMA-induced PGE2 expression in macrophage. The aim of this study was to evaluate the signal transduction pathways of BisGMA-induced PGE2 production in murine RAW264.7 macrophages.

**Methodology/Principal Findings:**

Herein, we demonstrate that BisGMA can exhibit cytotoxicity to RAW264.7 macrophages in a dose- and time-dependent manner (p<0.05). In addition, PGE2 production, COX-2 expression, and cPLA2 phosphorylation were induced by BisGMA on RAW264.7 macrophages in a dose- and time-dependent manner (p<0.05). Moreover, BisGMA could induce the phosphorylation of ERK1/2 pathway (MEK1/2, ERK1/2, and Elk), p38 pathway (MEK3/6, p38, and MAPKAPK2), and JNK pathway (MEK4, JNK, and c-Jun) in a dose- and time-dependent manner (p<0.05). Pretreatment with AACOCF3, U0126, SB203580, and SP600125 significantly diminished the phosphorylation of cPLA2, ERK1/2, p38, and JNK stimulated by BisGMA, respectively (p<0.05). BisGMA-induced cytotoxicity, cPLA2 phosphorylation, PGE2 generation, and caspases activation were reduced by AACOCF3, U0126, SB203580, and SP600125, respectively (p<0.05).

**Conclusions:**

These results suggest that BisGMA induced-PGE2 production may be *via* COX-2 expression, cPLA2 phosphorylation, and the phosphorylation of MAPK family. Cytotoxicity mediated by BisGMA may be due to caspases activation through the phosphorylation of cPLA2 and MAPKs family.

## Introduction

Bisphenol A-glycidyl-methacrylate (BisGMA) is synthesized from diglycidyl ether and methacrylic acid of bisphenol-A type epoxy resin [1]. The most commonly composite resins are composed of BisGMA monomers or its derivatives. BisGMA-based resins are used to restore hard tissue, such as teeth and bone. The advantages of BisGMA-based resins include higher modulus, less shrinkage, and lower diffusivity [2]. The commercial composite resins could release BisGMA into peripheral environment. BisGMA, incubated with water- or organic-based medium for 1 to 180 days, was leachable at a concentration range about 10^−3^ to 10^−1^ or 10^−1^ to 10 µM, respectively [3]. Yap et al. have purposed that the leachable BisGMA monomers may result in tissue damage through inflammatory reactions [4]. The activation of innate immune cells, especially macrophages, play a key regulator leading to inflammation [5]. Recently, we have demonstrated that BisGMA could induce cytotoxicity and genotoxicity in macrophages [6]. BisGMA could induce macrophage activation, such as the expression of surface antigens and the generation of proinflammatory mediators, including tumor necrosis factor (TNF)-α, interleukin (IL)-1β, IL-6 nitric oxide, and reactive oxygen species *via* the phosphorylation of PI3K/Akt, the degradation of IκB, and the activation of NFκB [7], [8].

Prostaglandin E2 (PGE2) is one of the pro-inflammatory mediators expressed at the site of tissue damage and stimulated by other proinflammatory cytokines such as TNF-α, IL-1β, and IL-6. PGE2 is a metabolite of arachidonic acid (AA) and is progressively produced by cytosolic phospholipase A2 (cPLA2), cyclooxygenases (COX), and PG synthases [9]. cPLA2 has been demonstrated to induce apoptosis through increased AA in *Mycobacterium tuberculosis*-infected human macrophages [10]. cPLA2 activation is regulated by mitogen-activated protein kinases (MAPKs), which are serine and thronine kinases, in macrophages [11], [12]. MAPKs, such as extracellular signal-regulated kinase (ERK) 1/2, p38, and c-JUN N-terminal kinase (JNK), can be activated by MAPK kinases (MEKs) and in turn phosphorylate and active the substrates. The MAPKs has been clearly identified three separate linear pathways including (1) ERK1/2 pathway: MEK1/2, ERK1/2, Elk; (2) p38 pathway: MEK3/6, p38, MAPKAPK2; (3) JNK pathway: MEK4, JNK, cJUN [13].

Previously, BisGMA was found to generate PGE2 *via* COX2 protein expression in human pulp cells [14]. However, the role of cPLA2 activation on BisGMA-induced PGE2 generation and cytotoxicity in macrophage still remains to be elucidated. In this study, the effects of BisGMA on murine macrophage RAW264.7 cells were determined through measuring the production of PGE2 by enzyme-linked immunosorbent assay (ELISA) and cytotoxicity. Western blot was used to evaluate COX-2 expression, the phosphorylation of cPLA2, and the phosphorylation of MAPKs family to clarify the signal transduction pathways.

## Materials and Methods

### Materials

Dulbecco’s modified Eagle’s medium (DMEM), fetal bovine serum (FBS), streptomycin and penicillin were obtained from Life Technologies (Grand Island, NY, USA). Enhanced chemiluminescence reagents were purchased from GE Healthcare (Piscataway, NJ, USA). PGE2 ELISA kit was obtained from eBiosciences (San Diego, CA, USA). Antibodies for COX-2, non-phosphorylation types of p38, cPLA2, MEK1/2, ERK1/2, Elk, MEK3/6, MAPKAPK2, MEK4, JNK, cJUN, phosphorylation types of cPLA2 (Ser505), MEK1/2 (Ser218/Ser222), ERK1/2 (Tyr204), Elk (Ser383), MEK3/6 (Ser189/Ser207), MAPKAPK2 (Thr222), MEK4 (Ser80), JNK (Thr183/Tyr185), cJUN (Ser63/73), and arachidonyl trifluoromethyl ketone (AACOCF3) were obtained from Santa Cruz Biotechnology (Santa Cruz, CA, USA). Antibodies for the phosphorylation type of p38 (Thr180/Tyr182) was purchased from Cell Signaling Technology (Danvers, MA, USA). Secondary antibodies were obtained from Jackson ImmunoResearch Laboratories (West Grove, PA, USA). 1,4-di-amino-2,3- dicyano-1,4-bis [2-amino-phenylthio] butadiene (U0126), 4-(4-fluorophenyl)-2-(4-methylsulfinyl-phenyl)-5-(4-pyridyl)-1Himidazole (SB203580), and Anthra(1,9-cd) pyrazol-6(2H)-one (SP600125) were obtained from Calbiochem-Novabiochem (La Jolla, CA, USA). Other chemicals were purchased from Sigma-Aldrich (St Louis, MO, USA). BisGMA was dissolved in dimethyl sulfoxide (DMSO). The final volume of DMSO added was lower than 0.5% which is a non-toxic concentration.

### Cell Culture

Murine macrophage cell line, RAW264.7, was obtained from Bioresource Collection and Research Center (BCRC 60001; Hsinchu, Taiwan). Cells were cultured in DMEM containing 10% FBS, 100 µg/ml streptomycin, and 100 U/ml penicillin. RAW 264.7 cells were maintained at sub-confluence in a 95% air and 5% CO_2_ humidified atmosphere at 37°C. To investigate the effects of BisGMA on RAW264.7 macrophages, cells were seeded on the plates and cultured for 24 h. After cell attachment, the medium were replaced with serum-free medium. Cells pretreated with or without 1 µM ERK1/2 inhibitor U0126, 30 µM p38 inhibitor SB203580, 30 µM JNK inhibitor SP600125, and 30 µM cPLA2 inhibitor AACOCF3 for 30 min. And then, RAW264.7 macrophages were incubated with different concentrations (0, 0.1, 0.3, 1, and 3 µM) of BisGMA for 15 min or 2 h.

### Cytotoxicity Assay

Cytotoxicity was measured by the release of lactate dehydrogenase (LDH). Briefly, 1×10^5^ cells were treated with various concentrations (0, 0.1, 0.3, 1, and 3 µM) of BisGMA for the indicated treatment period. Then, 1% Triton-X 100 was added to another well and incubated for 45 min. 50 µl supernatants of each well was transferred to new 96-well plate and added 50 µl of reconstituted substrate mix from CytoTox® 96 nonradioactive assay kit (Promega, Sunnyvale, CA, USA). After 30 min, the stop solution was added to each well. Absorbance was recorded at 490 nm by using a microplate reader (Dynatech MR 4000; Dynatech, Boston, MA, USA). Cell viability was calculated according to our perious study [6].

### Measurement of PGE2

The protein concentrations of PGE2 were measured by using ELISA [15]. Briefly, 5×10^4^ cells were incubated with different concentrations (0, 0.1, 0.3, 1, and 3 µM) of BisGMA for 2 h. The PGE2 levels in culture medium were measured by ELISA kit (eBiosciences, Diego, CA, USA) according to the manufacturer’s instructions.

### Western Blotting

To investigate the expression of COX2, phosphorylation and non-phosphorylation types of cPLA2, cells were treated with BisGMA for 2 h. To evaluate the phosphorylation and non-phosphorlation types of MEK1/2, ERK1/2, Elk, MEK3/6, p38, MAPKAPK2, MEK4, JNK, and cJUN, cells were treated with BisGMA for 15 min. After treatment, 50 µg of cell lysate protein, was separated by SDS-PAGE, and electrophoretically transferred to polyvinylidene difluoride membrane. The membranes were blocked with 5% (w/v) nonfat dried milk for 1 h at room temperature to reduce nonspecific binding, washed with PBS containing 0.1% Tween-20 (PBST), then probed with antibodies including COX-2, phosphorylation and non-phosphorylation of cPLA2, MEK1/2, ERK1/2, Elk, MEK3/6, p38, MAPKAPK2, MEK4, JNK, and cJUN. After membranes were washed again with PBST, a 1∶10,000 (v/v) dilution of horseradish peroxidase-labeled IgG was added at room temperature for 1 h, and the blots were developed using enhanced chemiluminescence reagents. The phosphorylation and expression of proteins were quantified with a LAS-4000 mini luminescent image analyzer (GE Healthcare Life Sciences, Tokyo, Japan) [16].

### Fluorometric Assay for Caspases Activities

Caspases activities were analyzed with a caspases fluorometric assay kits (Enzo Life Sciences, Plymouth Meeting, PA, USA) according to our recent study [6]. Briefly, cell lysates from each sample was mixed with a reaction buffer containing the fluorogenic substrates of caspase-3, -8, and -9, which are DEVD-AFC, IETD-AFC, and LEHD-AFC. The data were collected by using a fluorescence microplate reader (Molecular Devices, CA, USA) at excitation/emission wavelengths of 400/505 nm.

### Statistical Analysis

At least three independent experiments were performed as indicated in the figure legends. All data were expressed as mean ± standard deviation (SD). Statistical analysis was performed by using one-way analysis of variance (ANOVA) with Bonferroni-adjusted post hoc tests for multigroup comparisons; p<0.05 was considered significantly for each test.

## Results

### Effects of BisGMA on RAW 264.7 Macrophages

**Figure 1:**
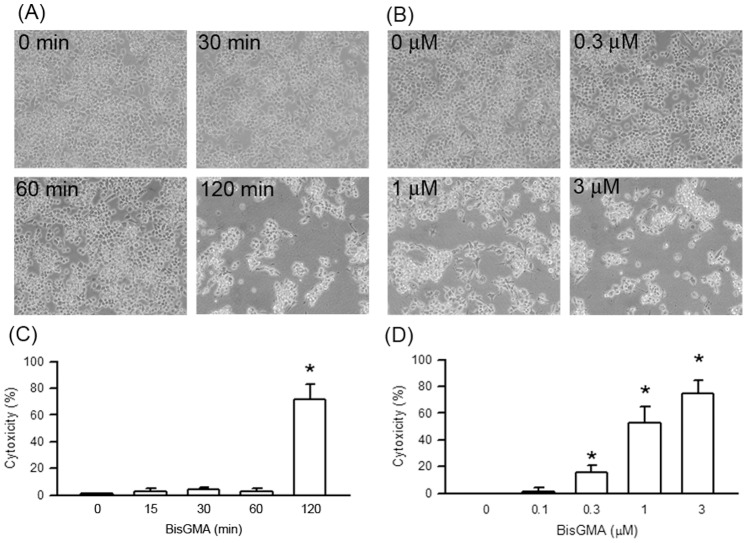
Cytotoxicity and effects of BisGMA on the morphology of RAW264.7 macrophages. (A) Cells were exposed to 3 µM BisGMA for the indicated treatment periods. (B) Cells were exposed to the indicated concentrations of BisGMA for 2 h. The morphology exchange were observed with an inverted microscope (Original magnification×200). (C) Cells were exposed to 3 µM BisGMA for the indicated treatment periods measured by LDH assay. (D) Cells were exposed to the indicated concentrations of BisGMA for 2 h. Values are expressed as mean ± SD (n = 5). *represents significant difference from control values with *p*<0.05.

The changes of RAW264.7 macrophage morphology and cell density after exposure to 3 µM BisGMA for the indicated treatment periods and the indicated concentrations of BisGMA for 2 h were shown in Fig. 1A and 1B, respectively. In addition, cytotoxicity of BisGMA was measured by the release of cytoplasmic enzyme LDH. As shown figures 1C and 1D, BisGMA exhibited cytotoxicity to RAW264.7 macrophage in a dose-dependent and a time-dependent manner (p<0.05), respectively.

### Effects of BisGMA on the Expression of PGE2 and COX-2

**Figure 2:**
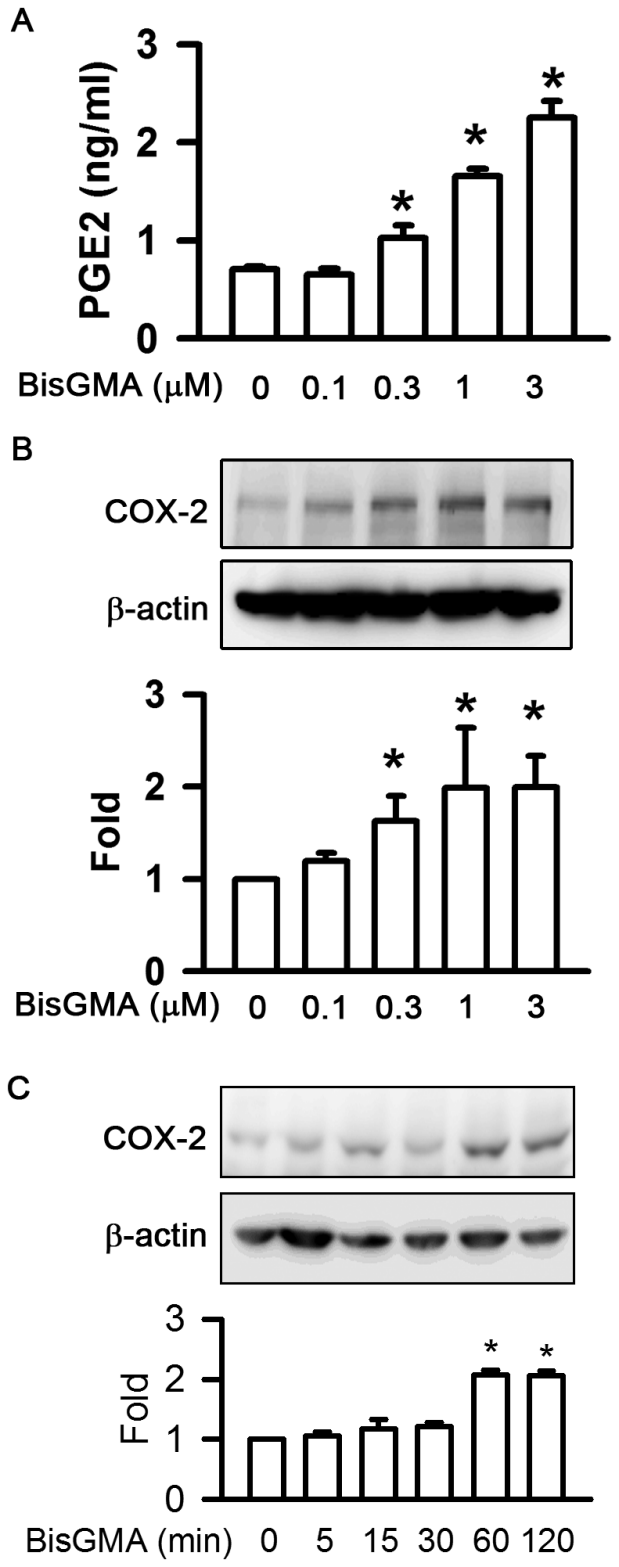
Effects of BisGMA on the expression of PGE2 and COX-2. (A) RAW264.7 macrophages were stimulated with the indicated concentrations of BisGMA for 2 h. The supernatant was harvested and detected by ELISA assay of PGE2. Values are expressed as mean ± SD (n = 3). *represents significant difference from control values with *p*<0.05. (B) Cells were exposed to the indicated concentrations of BisGMA for 2 h. Cells were harvested and protein extracts were subjected to SDS-PAGE. Values are expressed as mean ± SD (n = 3). The ratio of immunointensity between the COX-2 and β-actin was calculated. *represents significant difference from control values with *p*<0.05. (C) Cells were stimulated with 3 µM BisGMA for the indicated treatment periods. Cells were harvested and protein extracts were subjected to SDS-PAGE. Western blot analysis using antibodies against COX-2 and β-actin. Values are expressed as mean ± SD (n = 3). The ratio of immunointensity between the COX-2 and β-actin was calculated. *represents significant difference from control values with *p*<0.05.

The effect of BisGMA on the generation of PGE2 in RAW264.7 macrophages by ELISA assay was shown in figure 2A. The production of PGE2 was demonstrated in a dose-dependent manner (p<0.05). The levels of PGE2 were 0.71, 0.65, 1.02, 1.66, and 2.25 ng/ml at the concentrations of 0, 0.1, 0.3, 1, and 3 µM BisGMA for 2 h, respectively. As shown in figure 2B, BisGMA was demonstrated to increase COX-2 protein expression in a dose-dependent manner (p<0.05). The expressions of COX-2 were 1.00, 1.19, 1.62, 1.99, and 2.00 fold at the concentrations of 0, 0.1, 0.3, 1, and 3 µM BisGMA for 2 h, respectively. In addition, BisGMA was demonstrated to increase COX-2 protein expression in a time-dependent manner (p<0.05). The expressions of COX-2 were 1.00, 1.06, 1.17, 1.22, 2.07, and 2.07 fold incubated with 3 µM BisGMA for 0, 5, 15, 30, 60, and 120 min, respectively (Fig. 2C).

### Effects of BisGMA on the Phosphorylation of cPLA2

**Figure 3:**
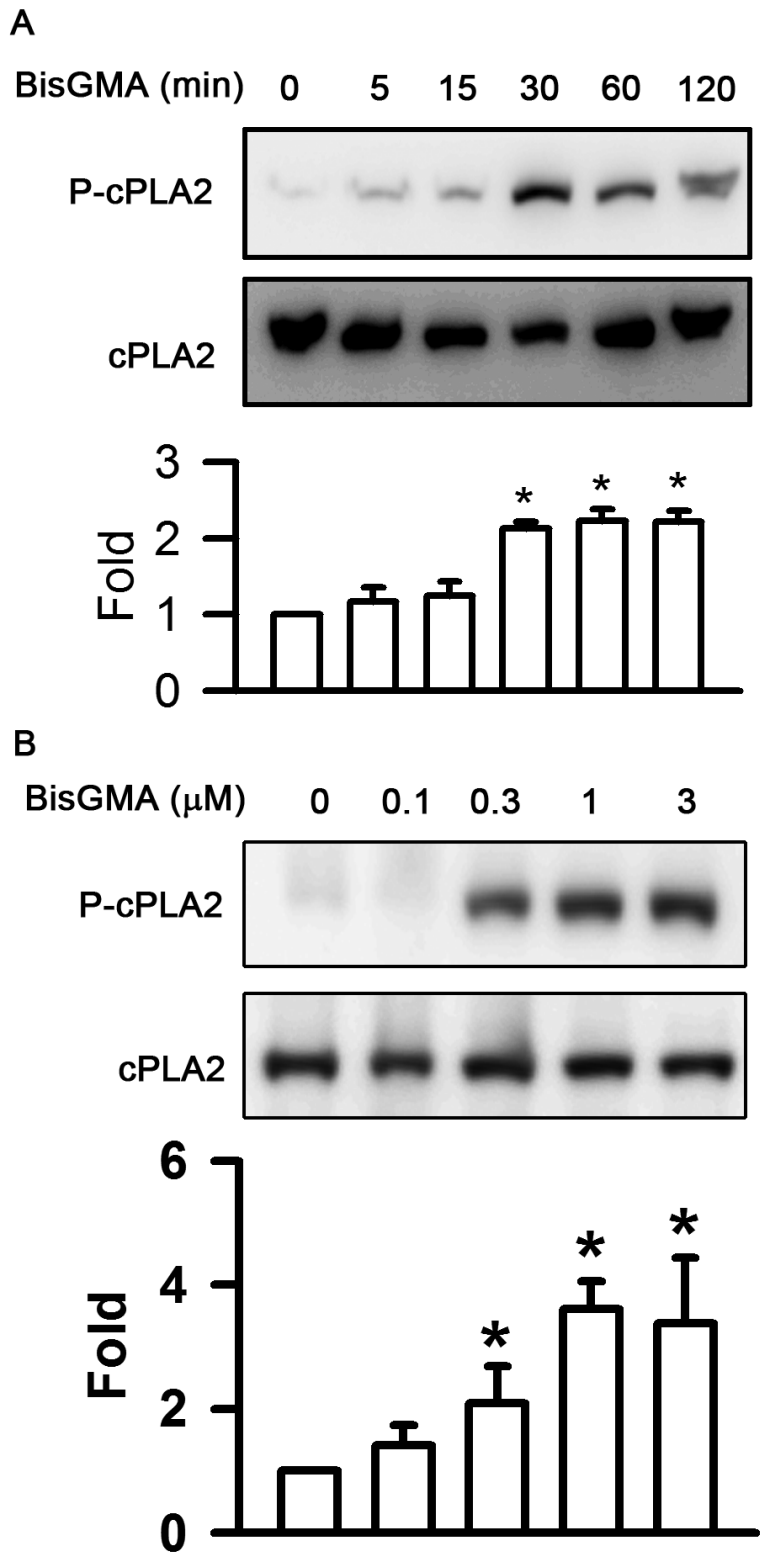
Effects of BisGMA on the phosphorylation of cPLA2. (A) Cells were stimulated with 3 µM BisGMA for the indicated treatment periods. (B) Cells were stimulated with the indicated concentrations of BisGMA for 2 h. Cells were harvested and protein extracts were subjected to SDS-PAGE. Western blot analysis using antibodies against phosphorylated and total cPLA2. The ratio of immunointensity between the phosphorylation and total protein was calculated. Values are expressed as mean ± SD (n = 3). *represents significant difference from control values with *p*<0.05.

The exposure of RAW264.7 macrophages to BisGMA evoked the phosphorylation of cPLA2 in a time-dependent manner (p<0.05) (Fig. 3A). The duration of 3 µM BisGMA treatment longer than 30 min significantly upregulated the phosphorylation of cPLA2 (p<0.05). The levels of cPLA2 phosphorylation were 1.00, 1.16, 1.25, 2.14, 2.23, and 2.22 fold after incubation with 3 µM BisGMA for 0, 5, 15, 30, 60, and 120 min, respectively. The exposure of RAW264.7 macrophages to BisGMA also evoked the phosphorylation of cPLA2 in a concentration-dependent manner (p<0.05) (Fig. 3B). The concentration of BisGMA higher than 0.3 µM significantly upregulated the phosphorylation of cPLA2 (p<0.05). The levels of cPLA2 phosphorylation were 1.00, 1.41, 2.09, 3.61, and 3.38 fold at the concentrations of 0, 0.1, 0.3, 1, and 3 µM BisGMA for 15 min, respectively.

### Effects of AACOCF3 on BisGMA-induced cPLA2 Phosphorylation, COX-2 Expression and PGE2 Generation

**Figure 4:**
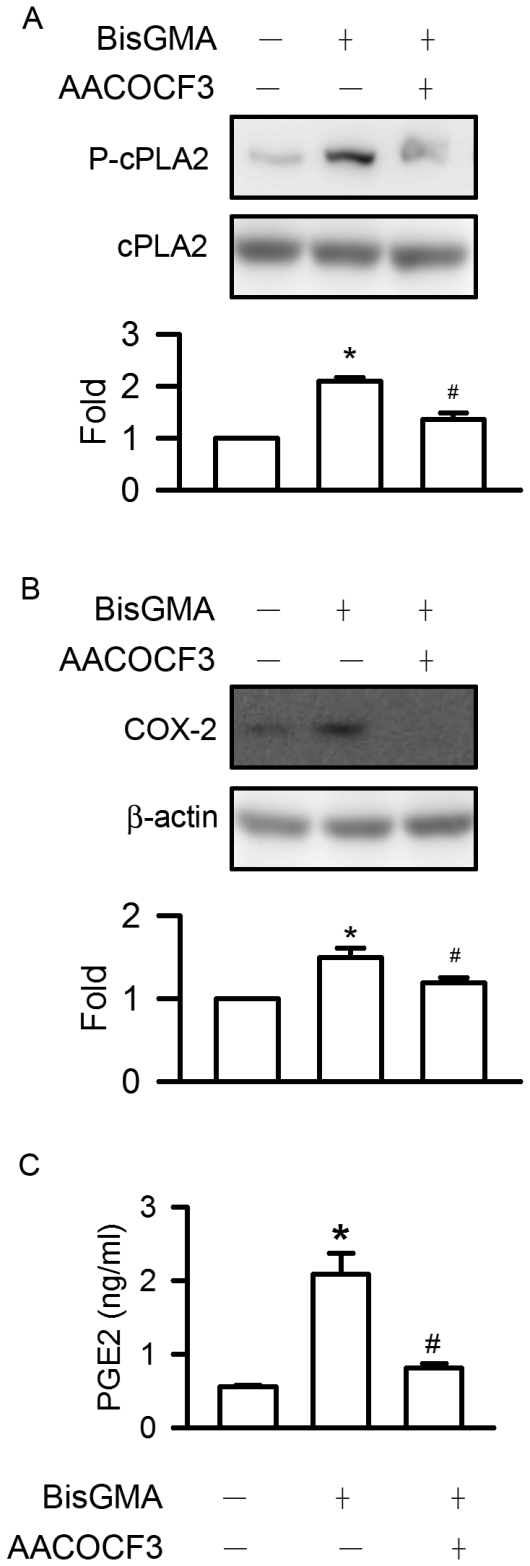
Effects of AACOCF3 on the cPLA2 phosphorylation, COX-2 expression, and PGE2 generation. After treatment with 30 µM AACOCF3 for 30 min, RAW264.7 macrophages were stimulated with 3 µM BisGMA for 2 h. Cells were harvested and protein extracts were subjected to SDS-PAGE. Western blot analysis using antibodies against (A) phosphorylated and total cPLA2; (B) COX-2 and β-actin. The ratio of immunointensity between the phosphorylation and total protein was calculated. Values are expressed as mean ± SD (n = 3). *represents significant difference from control values with *p*<0.05. (C) The supernatant was harvested and detected by ELISA assay of PGE2. Values are expressed as mean ± SD (n = 3). *represents significant difference from control values with *p*<0.05.

Pretreatment of AACOCF3 significantly diminished BisGMA-induced cPLA2 phosphorylation from 2.09 to 1.36 fold (p<0.05) (Fig. 4A). Pretreatment of AACOCF3 significantly decreased BisGMA-induced COX-2 expression from 1.50 to 1.19 fold (p<0.05) (Fig. 4B). Pretreatment of AACOCF3 significantly downregulated BisGMA-induced PGE2 generation from 2.09 to 0.56 ng/ml (p<0.05) (Fig. 4C).

### Effects of BisGMA on the Phosphorylation of ERK1/2 Pathway

**Figure 5:**
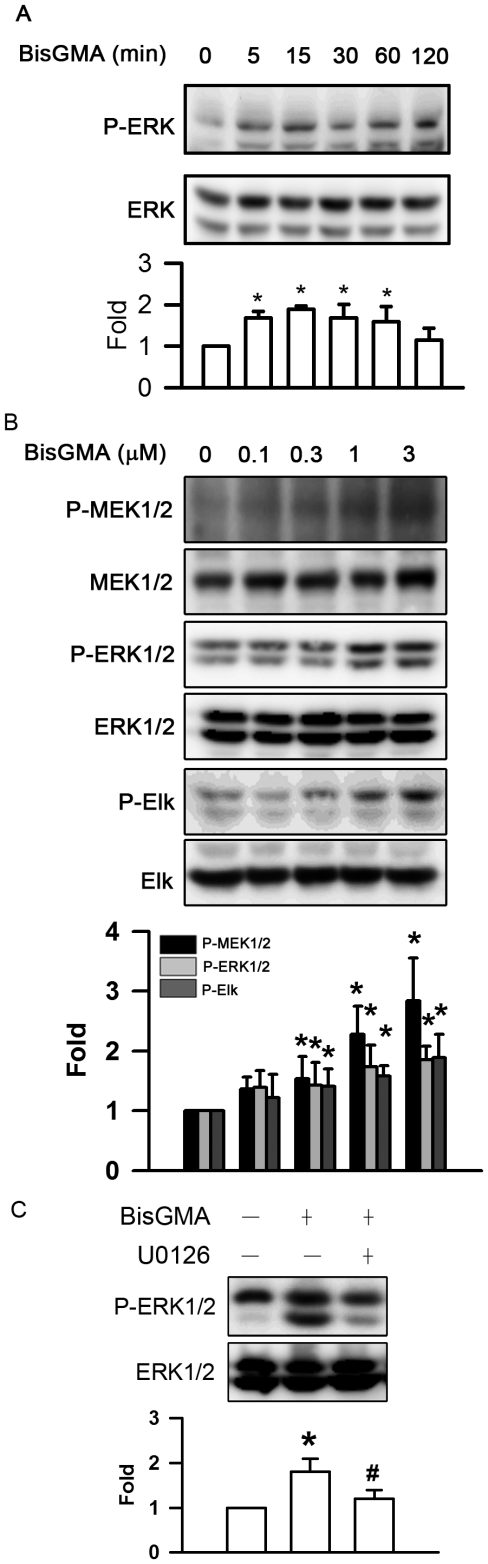
Effects of BisGMA on the phosphorylation of ERK1/2 pathway. (A) RAW264.7 macrophages were stimulated with 3 µM BisGMA for the indicated treatment periods. Cells were harvested and protein extracts were subjected to SDS-PAGE. Western blot analysis using antibodies against phosphorylated and total ERK1/2. (B) Cells were stimulated with the indicated concentrations BisGMA for 15 min. (C) After treatment with 1 µM U0126 for 30 min, cells were stimulated with 3 µM BisGMA for 15 min. Cells were harvested and protein extracts were subjected to SDS-PAGE Western blot analysis using antibodies against the phosphorylated and total MEK1/2, ERK1/2, and Elk. The ratio of immunointensity between the phosphorylation and total protein was calculated. Values are expressed as mean ± SD (n = 3). *represents significant difference from control values with *p*<0.05. #represents significant difference from BisGMA values with *p*<0.05.

The exposure of RAW264.7 cells to BisGMA evoked the phosphorylation of ERK1/2 in a time-dependent manner (p<0.05) (Fig. 5A). The duration of 3 µM BisGMA treatment between 5 min to 60 min significantly upregulated the phosphorylation of ERK1/2 (p<0.05). The levels of ERK1/2 phosphorylation were 1.00, 1.68, 1.89, 1.68, 1.60, and 1.16 fold after incubation with 3 µM BisGMA for 0, 5, 15, 30, 60, and 120 min, respectively. As shown in Fig. 5B, BisGMA was found to induce the phosphorylation of MEK1/2, ERK1/2, and Elk in a dose-dependent manner (p<0.05). The phosphorylated levels of MEK1/2 were 1.00, 1.35, 1.61, 2.28, and 2.84 fold at the concentrations of 0, 0.1, 0.3, 1, and 3 µM BisGMA for 15 min, respectively. The phosphorylated levels of ERK1/2 were 1.00, 1.39, 1.43, 1.73, and 1.86 fold at the concentrations of 0, 0.1, 0.3, 1, and 3 µM BisGMA for 15 min, respectively. The phosphorylated levels of Elk were 1.00, 1.22, 1.41, 1.58, and 1.89 fold at the concentrations of 0, 0.1, 0.3, 1, and 3 µM BisGMA for 15 min, respectively. Furthermore, pretreatment with 1 µM ERK1/2 inhibitor U0126 for 30 min significantly diminished BisGMA-stimulated phosphorylation of ERK1/2 from 1.8 to 1.2 fold (p<0.05) (Fig. 5C).

### Effects of BisGMA on the Phosphorylation of p38 Pathway

**Figure 6:**
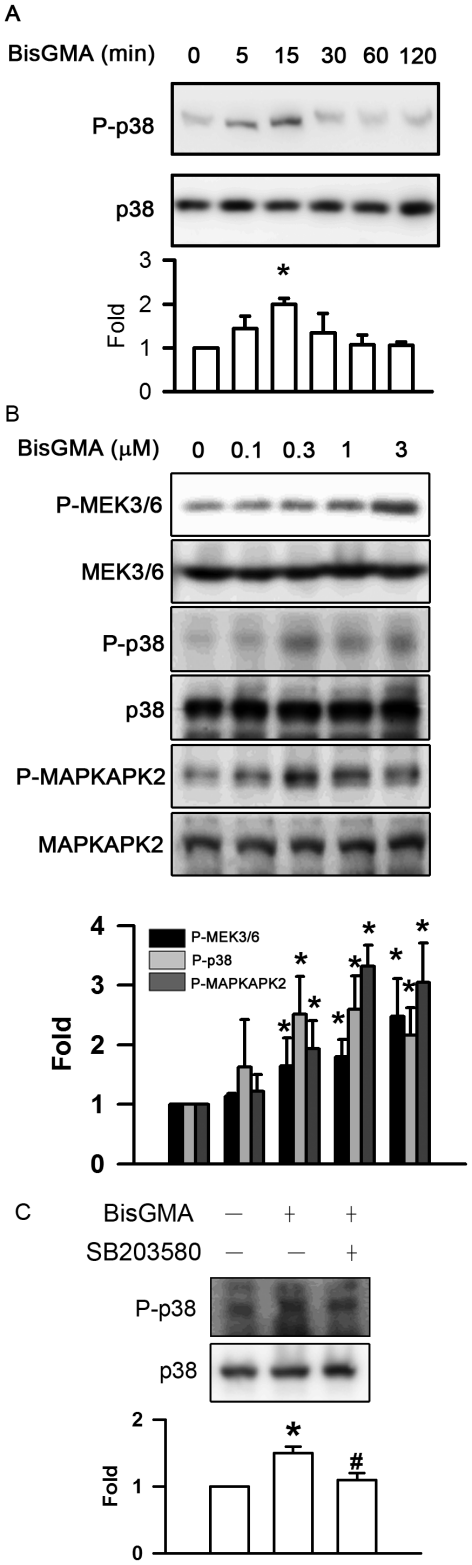
Effects of BisGMA on the phosphorylation of p38 pathway. (A) RAW264.7 macrophages were stimulated with 3 µM BisGMA for the indicated treatment periods. Cells were harvested and protein extracts were subjected to SDS-PAGE. Western blot analysis using antibodies against the phosphorylated and total p38. (B) Cells were stimulated with the indicated concentrations of BisGMA for 15 min. (C) After treatment with 30 µM SB203580 for 30 min, cells were stimulated with 3 µM BisGMA for 15 min. Cells were harvested and protein extracts were subjected to SDS-PAGE Western blot analysis using antibodies against phosphorylated and total MEK3/6, p38, and MAPKAPK-2. The ratio of immunointensity between the phosphorylation and total protein was calculated. Values are expressed as mean ± SD (n = 3). *represents significant difference from control values with *p*<0.05. #represents significant difference from BisGMA values with *p*<0.05.

The exposure of RAW264.7 cells to BisGMA evoked the phosphorylation of p38 in a time-dependent manner (p<0.05) (Fig. 6A). 3 µM BisGMA treatment for 15 min significantly upregulated the phosphorylation of p38 (p<0.05). The levels of p38 phosphorylation were 1.00, 1.44, 2.00, 1.35, 1.08, and 1.06 fold after incubation with 3 µM BisGMA for 0, 5, 15, 30, 60, and 120 min, respectively. As shown in figure 6B, BisGMA was found to induce phosphorylation of MEK3/6, p38, MAPKAPK2 in a dose-dependent manner (p<0.05). The phosphorylated levels of MEK3/6 were 1.00, 1.14, 1.64, 1.79, and 2.48 fold at the concentrations of 0, 0.1, 0.3, 1, and 3 µM BisGMA for 15 min, respectively. The phosphorylated levels of p38 were 1.00, 1.62, 2.51, 2.60, and 2.17 fold at the concentrations of 0, 0.1, 0.3, 1, and 3 µM BisGMA for 15 min, respectively. The phosphorylated levels of MAPKAPK2 were 1.00, 1.22, 1.94, 3.32, and 3.05 fold at the concentrations of 0, 0.1, 0.3, 1, and 3 µM BisGMA for 15 min, respectively. In addition, the pretreatment with 30 µM p38 inhibitor SB203580 for 30 min significantly diminished BisGMA-stimulated phosphorylation of p38 from 1.5 to 1.1 fold (p<0.05) (Fig. 6C).

### Effects of BisGMA on the Phosphorylation of JNK Pathway

**Figure 7:**
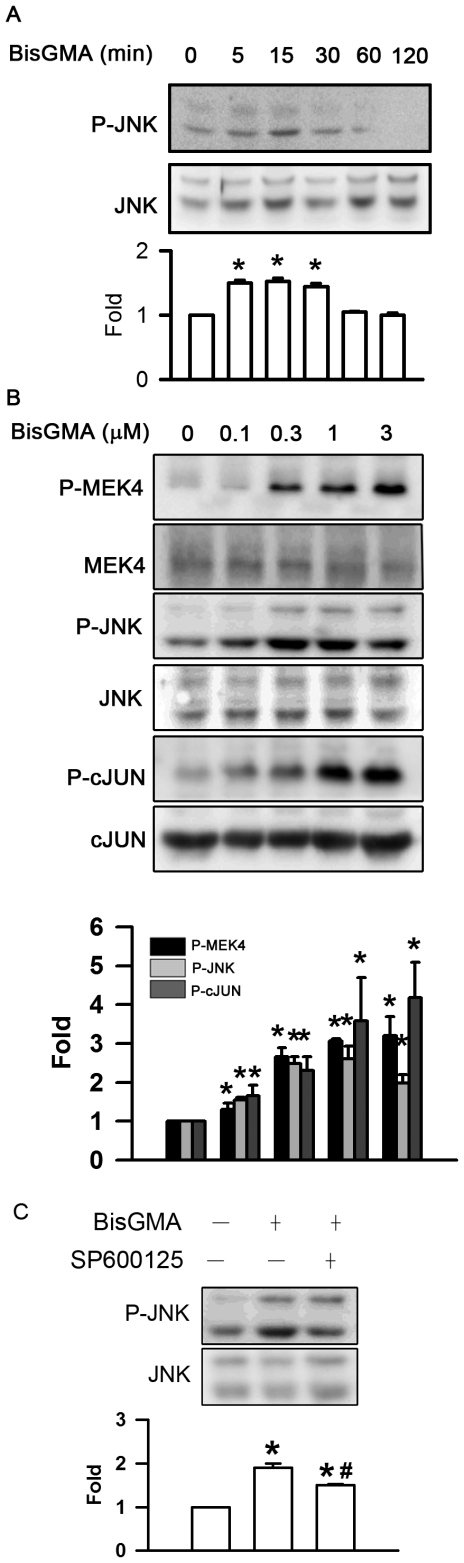
Effects of BisGMA on the phosphorylation of JNK pathway. (A) RAW264.7 macrophages were stimulated with 3 µM BisGMA for the indicated treatment periods. Cells were harvested and protein extracts were subjected to SDS-PAGE. Western blot analysis using antibodies against phosphorylated and total JNK. (B) Cells were stimulated with the indicated concentrations of BisGMA for 15 min. (C) After treatment with 30 µM SP600125 for 30 min, cells were stimulated with 3 µM BisGMA for 15 min. Cells were harvested and protein extracts were subjected to SDS-PAGE Western blot analysis using antibodies against phosphorylated and total MEK4, JNK, and cJUN. The ratio of immunointensity between the phosphorylation and total protein was calculated. Values are expressed as mean ± SD (n = 3). *represents significant difference from control values with *p*<0.05. #represents significant difference from BisGMA values with *p*<0.05.

The exposure of RAW264.7 cells to BisGMA evoked the phosphorylation of JNK in a time-dependent manner (p<0.05) (Fig. 7A). The duration of 3 µM BisGMA treatment between 5 min to 30 min significantly upregulated the phosphorylation of JNK (p<0.05). The levels of JNK phosphorylation were 1.00, 1.50, 1.53, 1.45, 1.05, and 1.00 fold incubated with 3 µM BisGMA for 0, 5, 15, 30, 60, and 120 min, respectively. BisGMA was found to induce the phosphorylation of MEK4, JNK, c-Jun in a dose-dependent manner (p<0.05) (Fig. 7B). The phosphorylated levels of MEK4 were 1.00, 1.30, 2.66, 3.06, and 3.20 fold at the concentrations of 0, 0.1, 0.3, 1, and 3 µM BisGMA for 15 min, respectively. The phosphorylated levels of JNK were 1.00, 1.55, 2.51, 2.60, and 2.17 fold at the concentrations of 0, 0.1, 0.3, 1, and 3 µM BisGMA for 15 min, respectively. The phosphorylated levels of c-Jun were 1.00, 1.65, 1.94, 3.32, and 3.05 fold at the concentrations of 0, 0.1, 0.3, 1, and 3 µM BisGMA for 15 min, respectively. Pretreatment with 30 µM JNK inhibitor SP600125 for 30 min significantly diminished BisGMA-stimulated phosphorylation of JNK from 1.9 to 1.5 fold (p<0.05) (Fig. 7C).

### Effects of U0126, SB203580, SP600125 on BisGMA-induced cPLA2 Phosphorylation and PGE2 Generation

**Figure 8:**
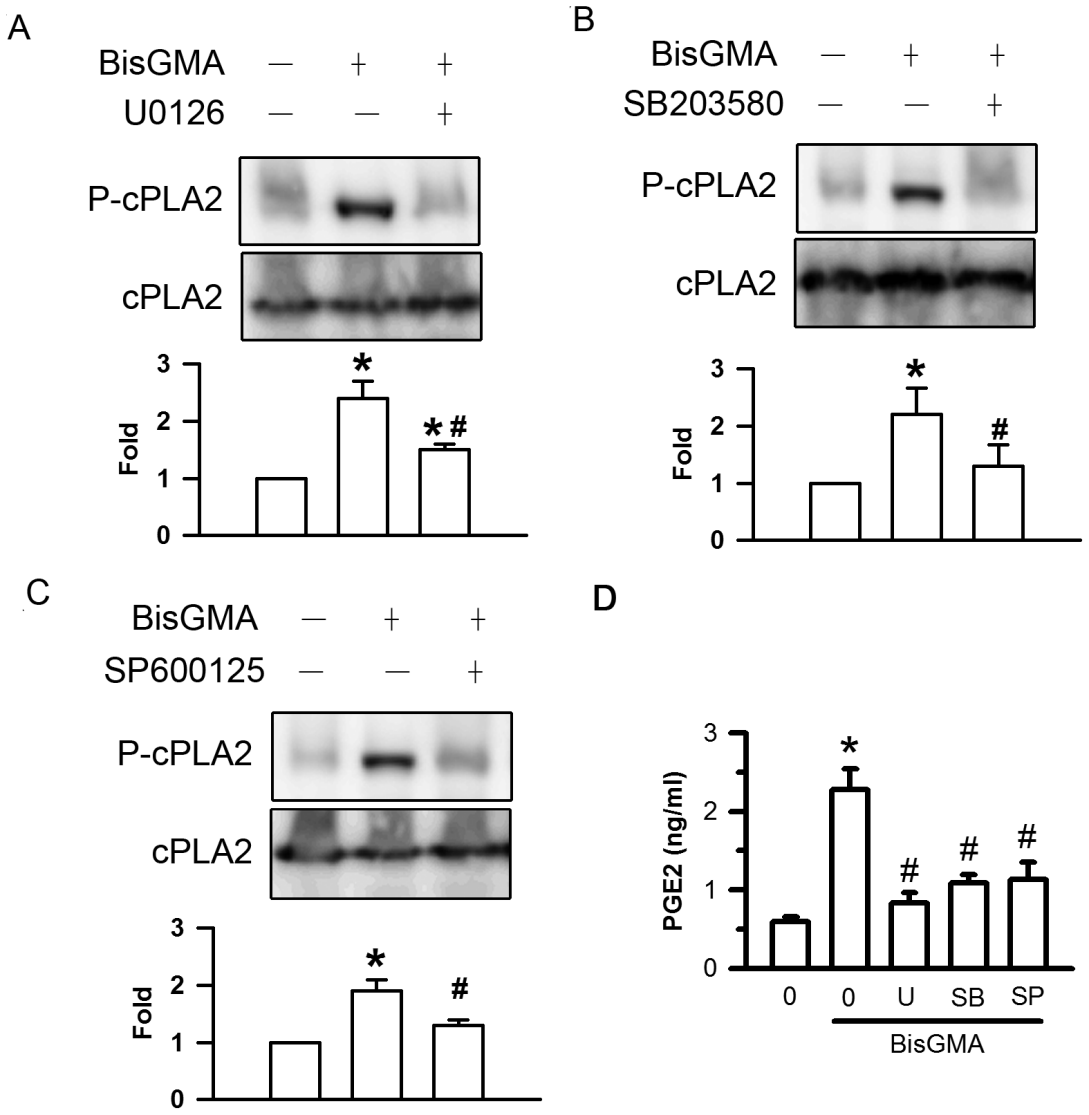
Effects of U0126, SB203580, and SP600125 on BisGMA-induced cPLA2 phosphorylation and PGE2 generation. After treatment with 1 µM U0126 (A), 30 µM SB203580 (B), and 30 µM SP600125 (C) for 30 min, RAW264.7 macrophages were stimulated with 3 µM BisGMA for 2 h. Cells were harvested and protein extracts were subjected to SDS-PAGE Western blot analysis using antibodies against the phosphorylated and total ERK1/2, p38, and JNK. The ratio of immunointensity between the phosphorylation and total protein was calculated. (D) The supernatant was harvested and detected by ELISA assay of PGE2. (D) The supernatant was harvested and detected by ELISA assay of PGE2. Values are expressed as means ± S.D. (*n = *3 in each group). *represents significant difference from control values with *p*<0.05. #represents significant difference from BisGMA values with *p*<0.05.

Pretreatment of U0126 significantly diminished BisGMA-stimulated phosphorylation of cPLA2 from 2.4 to 1.5 fold (p<0.05) (Fig. 8A). Pretreatment with SB203580 significantly decreased BisGMA-stimulated phosphorylation of cPLA2 from 2.2 to 1.3 fold (p<0.05) (Fig. 8B). Pretreatment with SP600125 significantly downregulated BisGMA-stimulated phosphorylation of cPLA2 from 1.9 to 1.3 fold. (p<0.05) (Fig. 8C). Pretreatment of U0126, SB203580, and SP600125 significantly diminished BisGMA-induced PGE2 generation from 2.28 to 0.83, 1.09, and 1.13 ng/ml, respectively (p<0.05) (Fig. 8D).

### Effects of U0126, SB203580, SP600125, and AACOCF3 on BisGMA-induced Caspases 3, 8, and 9 Activations and Cytotoxicity

**Figure 9:**
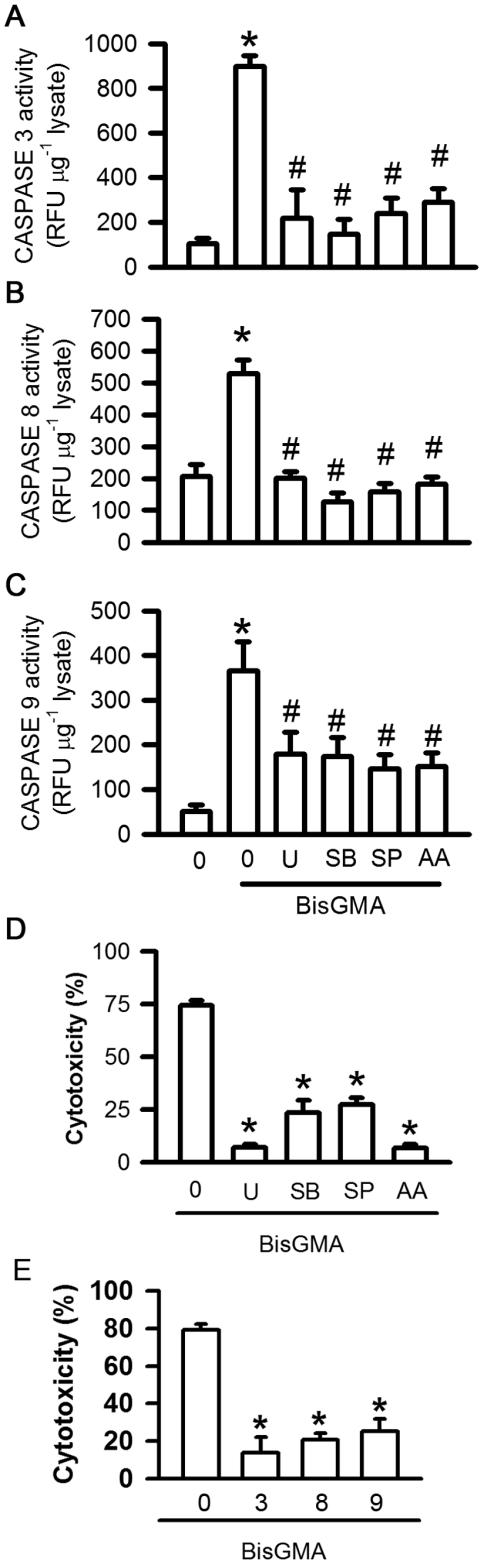
Effects of U0126, SB203580, SP600125, and AACOCF3 on BisGMA-induced caspases activation and cytotoxicity. After treatment with 1 µM U0126, 30 µM SB203580, 30 µM SP600125, and 30 µM AACOCF3 for 30 min, RAW264.7 macrophages were stimulated with 3 µM BisGMA for 2 h. Caspase 3 (A), 8 (B), and 9 (C) were measured by caspases assay kits. (D) Cytotoxicity was measured by MTT assay. (E) After treatment with 10 µM caspases 3 inhibitor (Z-DEVD-FMK), caspases 8 inhibitor (Z-IETD-FMK), and caspases 9 inhibitor (Z-LEHTD-FMK) for 30 min, cells were stimulated with 3 µM BisGMA for 2 h. Cytotoxicity was measured by MTT assay. Values are expressed as mean ± SD (n = 5). *represents significant difference from control values with *p*<0.05.

Pretreatment of U0126, SB203580, SP600125, and AACOCF3 significantly diminished BisGMA-induced caspase 3 activation from 6289.07 to 1532.79, 1027.51, 1674.3, and 2038.10 RFU/µg lysate, respectively (p<0.05) (Fig. 9A). Pretreatment of U0126, SB203580, SP600125, and AACOCF3 significantly decreased BisGMA-induced caspase 8 activation from 2119.10 to 805.33, 506.13, 636.22, and 728.33 RFU/µg lysate, respectively (p<0.05) (Fig. 9B). Pretreatment of U0126, SB203580, SP600125, and AACOCF3 significantly downregulated BisGMA-induced caspase 9 activation from 2563.17 to 1258.32, 1215.45, 1020.46, and 1058.54 RFU/µg lysate, respectively (p<0.05) (Fig. 9C). Pretreatment of U0126, SB203580, SP600125, and AACOCF3 significantly diminished BisGMA-induced cytotoxicity from 74.50 to 7.23, 23.66, 27.51, and 6.72%, respectively (p<0.05) (Fig. 9D). Pretreatment of caspase 3, 8, and 9 inhibitors significantly reduced BisGMA-induced cytotoxicity from 79.08 to 13.75, 20.85, and 25.31%, respectively (p<0.05) (Fig. 9E).

## Discussion

BisGMA is the most common used resin monomer for dental materials such as bonding agents, sealants, cement, bite guards, and dentures. To restore the caries cavity on the teeth bottom, the unpolymerized monomers of BisGMA can leach into oral cavity and prompt pulpal imflammation, cytoxicity, and delay pulpal healing [4], [17], [18]. The concentrations of BisGMA has been reported to leach into peripheral environment from 0.001 to 10 µM [3], thus we employed the doses of BisGMA from 0.1 to 3 µM in the present study. BisGMA was found to induce cytotoxicity at concentration of 0.3 µM in RAW264.7 macrophages. Similar results were found in human pulp cells [18] and macrophages [6]. Pro-inflammatory mediators, such as nitric oxide, reactive oxygen species, TNF-α, IL-1β, and IL-6, generation from macrophages are induced by lipopolysaccharide, interferon-γ, and BisGMA [7], [8], [19]. The moderate amount of proinflammatory mediators could kill or remove invasive microorganisms. However, over-expression of proinflammatory mediators would led to peripheral tissue damage and self-annihilation [20]. Macrophage activation is a hallmark of local inflammatory response. Previously, the simplified etch-and-rinse adhesive system, which contains BisGMA, could induce cytotoxicity and NO generation in alveolar macrophages [21]. In cell culture system, RAW264.7 macrophages have been used for the experiments of immunological function directly and as well as human U937 macrophages and peripheral boold mononuclear cells [22], [23]. Therefore, we chose RAW264.7 macrophages to evaluate the effects cytotoxicitiy, generation of PGE2, activation of MAPKs and cPLA2 in this study.

PGE2 plays an important role in acute and chronic inflammation, infection, pain, fever, and cancer [9], [24]. Macrophages activated by physiological or pathological stimuli could release PGE2 which increases vascular permeability, infiltration of neutrophils, macrophages, and mast cells into secretion sites [9]. COX is the rate-limiting enzyme that catalyzes the conversion of AA to PGE2. COX-2 is an inducible enzyme predominantly expressed in inflamed tissues [25]. In the present study, BisGMA was demonstrated to significantly evoke PGE2 secretion and COX-2 expression in RAW264.7 macrophages. Similar results were found in human pulp cells by Chang et al. [14]. Nuclear factor (NF)κB is an important transcription factor in the expression of COX-2 [26]. In perivous study, we have demonstrated BisGMA induced NFκB phosphorylation [7]. These results suggest that PGE2 generation was induced by BisGMA stimulated COX-2 expression *via* NFκB phosphorylation.

cPLA2 catalyzes the first step in hydrolysis of membrane phosphatidylcholine convert into AA, which is the precursor of proinflammatory lipid mediators such as PGs, thromboxane, and leukotrienes [27]. AA has been shown to induce chemotaxis, reactive oxygen species generation, and apototsis in neutrophils, epithelial cells, and macrophages [10], [28]. In addition, cPLA2 and AA directly associate to COX-2-derived PGE2 synthesis correlation with inflammation [29]. To the best of our knowledge, we first found the phosphorylation of cPLA2 induced by BisGMA in RAW264.7 macrophages. The phosphorylation of cPLA2 and secretion of PGE2 by BisGMA exhibited apparently in a parallel concentration-dependent manner. Pretreatment with cPLA2 inhibitor AACOCF3 suppressed cPLA2 phosphorylation, COX-2 expression, PGE2 generation, and cytotoxicity in BisGMA-stimulated RAW264.7 macrophages. These results indicated that the secretion of PGE2 and cytotoxicity induced by BisGMA may be *via* the phosphorylation of cPLA2.

Several studies have demonstrated the activation and phosphorylation of cPLA2 regulated by MAPKs in macrophage during stimulation [12], [30]. In addition, MAPKs play an important role in the stimulation of pro-inflammatory genes and mediators in macrophages [31]. MAPKs pathways are organized by three-tiered hierarchical kinases module. MAPKs are activated by MEKs, which is dual phosphorylation of threonine and tyrosine residues in the Thr-X-Tyr motif. MEK is activated by MEKs kinases (MEKKs) through serine and threonine phosphorylation [32]. ERK1/2 is activated and phosphorylated by MEK1/2. MEK1/2 is in turn regulated by Raf. The substrates of ERK1/2 are Elk and p90^RSK^. p38 is activated and phosphorylated by MEK3/6. MEK3/6 is in turn regulated by MEKK1/2/3/4. The substrate of p38 is MAPKAPK2 and ATF2. JNK is activated and phosphorylated by MEK4/7. MEK4/7 is in turn regulated by MEKK1/2/3/4. The substrate of JNK is cJUN [33]. In human pulp cells, BisGMA was reported to induce the phosphorylation of MEK1/2-ERK1/2 signaling and the production of PGE2 [14]. In the present study, we first purposed that the phosphorylation of MEK1/2, ERK1/2, and Elk was triggered by BisGMA in RAW264.7 macrophages. Pretreatment with U0126 inhibited BisGMA-induced phosphorylation of cPLA2, generation of PGE2, and cytotoxicicty. In addition, we also first found BisGMA-induced the phosphorylation of p38 pathway, including MEK3/6, p38, and MAPKAPK2, and JNK pathway, including MEK4, JNK, and cJUN. The MAPKs inhibitors U0126, SB203580, and SP600125 also significantly inhibited BisGMA-induced the phosphorylation of cPLA2 and generation of PGE2. Taken together, these results indicated that cPLA2 could participate in BisGMA-induced PGE2 generation in RAW 264.7 macrophages *via* MAPKs signal transduction pathways.

Recently, we demonstrated that BisGMA-induced cytotoxicity and apoptosis may be via caspases 3, 8, and 9 activation in RAW264.7 macrophages [6]. Previously, studies have shown that BisGMA-induced cytotoxicity may be *via* the generation of reactive oxygen species and the expression of hemeoxygenase-1 and carboxylesterase could against BisGMA-induced cytotoxicity in human pulp cells [18], [34]. cPLA2 has demonstrated to participate caspases activation and result in macrophage apoptosis [10]. Pretreatment with AACOCF3, U0126, SB203580, and SP600125 could reduce BisGMA-induced cytotoxicity. Furthermore, AACOCF3, U0126, SB203580, and SP600125 could suppress BisGMA-induced caspase 3, 8, and 9 activation. These results indicated that cPLA2 may participate in BisGMA-induced cytotoxicity and caspases activation in RAW 264.7 macrophages via MAPKs signal transduction pathways.

**Figure 10:**
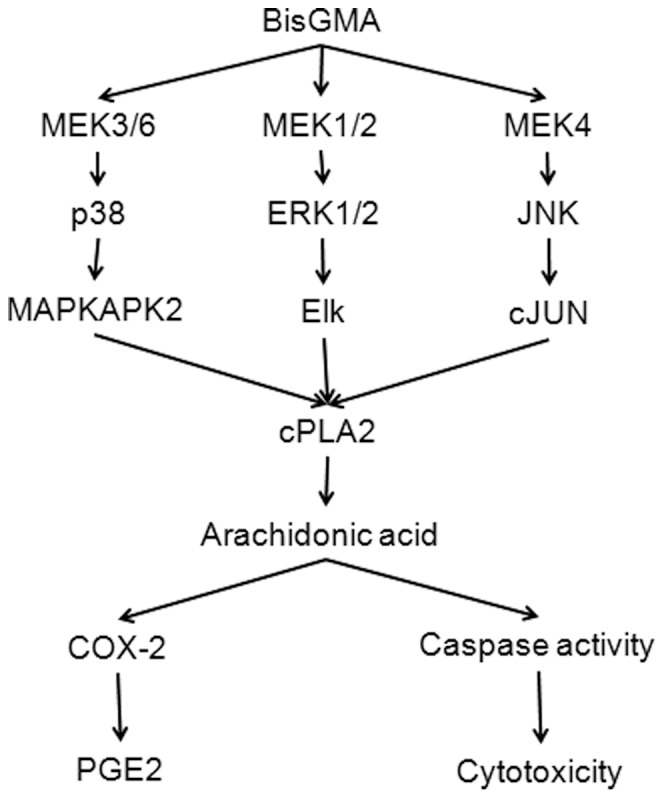
Schematic diagram illustrating the signaling pathways involved in BisGMA-induced PGE2 generation in RAW264.7 macrophages.

This study has investigated the mechanism of PGE2 generation and cytotoxicity induced by BisGMA in RAW264.7 macrophages (Fig. 10). The principal findings are as follows. BisGMA could effectively induce PGE2 production *via* COX-2 expression and cPLA2 phosphorylation. BisGMA induced the activation and phosphorylaion of p38 pathway, including MEK3/6, p38, and MAPKAPK2. BisGMA also induced the activation and phosphorylaion of ERK1/2 pathway, including MEK1/2, ERK1/2, and Elk. In addition, BisGMA induced the activation and phosphorylaion of JNK pathway, including MEK4, JNK, and cJUN. Moreover, U0126, SB203580, SP600125, and AACOCF3 inhibited BisGMA-induced cPLA2 phosphorylation, PGE2 generation, cytotoxicity, and caspases activation. In conclusion, we have shown that the generation of PGE2 and caspases activation induced by BisGMA may involve the phosphorylation of cPLA2 *via* ERK1/2, p38, and JNK pathways in RAW264.7 macrophages.
